# Validation study of 3D-printed anatomical models using 2 PLA printers for preoperative planning in trauma surgery, a human cadaver study

**DOI:** 10.1007/s00068-018-0970-3

**Published:** 2018-06-11

**Authors:** Lars Brouwers, Arno Teutelink, Fiek A. J. B. van Tilborg, Mariska A. C. de Jongh, Koen W. W. Lansink, Mike Bemelman

**Affiliations:** 1 Network Emergency Care Brabant, Elisabeth-Tweesteden Hospital, Hilvarenbeekseweg 60, 5022 GC Tilburg, The Netherlands; 2 Traumasurgery, Bernhoven Hospital, Nistelrodeseweg 10, 5406 PT Uden, The Netherlands; 3 Department of Radiology, Elisabeth-Tweesteden Hospital, Hilvarenbeekseweg 60, 5022 GC Tilburg, The Netherlands; 4 Clinical Epidemiologist, Brabant Trauma Registry, Network Emergency Care Brabant, Elisabeth-Tweesteden Hospital, Hilvarenbeekseweg 60, 5022 GC Tilburg, The Netherlands; 5 Traumasurgery, Elisabeth-Tweesteden Hospital, Hilvarenbeekseweg 60, 5022 GC Tilburg, The Netherlands

**Keywords:** 3D printing, Complex fractures, PLA, Human cadaver, Validation, Trauma surgery

## Abstract

**Introduction:**

3D printing contributes to a better understanding of the surgical approach, reduction and fixation of complex fractures. It is unclear how a 3D-printed model relates to a human bone. The accuracy of 3D-printed models is important to pre-bend plates and fit of surgical guides. We conduct a validation study in which we compare human cadavers with 3D-printed models to test the accuracy of 3D printing.

**Methods:**

Nine specimens were scanned, volume rendered into 3D reconstructions and saved as STL data. All models were in a ratio of 1:1 printed on the Ultimaker 3 and Makerbot Replicator Z18. Two independent observers measured all distances between the K-wires on the human cadavers, 2DCT, 3D reconstruction, Meshlab and both printers. A paired Samples *T* test was used to compare the measurements between the different modalities.

**Results:**

The least decrease in average distance in millimetres was seen in “the 3D printed pelvis 1”, − 0.3 and − 0.8% on respectively the Ultimaker and Makerbot when compared with cadaver Pelvis (1) The 3D model of “Hand 2” showed the most decrease, − 2.5 and − 3.2% on the Ultimaker and Makerbot when compared with cadaver hand (2) Most significant differences in measurements were found in the conversion from 3D file into a 3D print and between the cadaver and 3D-printed model from the Makerbot.

**Conclusion:**

Our 3D printing process results in accurate models suitable for preoperative workup. The Ultimaker 3 is slightly more accurate than the Makerbot Replicator Z18. We advise that medical professionals should perform a study that tests the accuracy of their 3D printing process before using the 3D-printed models in medical practice.

## Introduction

Complex fractures are difficult to characterise and analyse preoperatively, even with computed tomography (CT) [1, 3]. Surgeons generally need years of practice to transform a two-dimensional (2D) image into a three-dimensional (3D) image in their mind in order to get a proper understanding of the fracture patterns. CT software however easily enables volume rendering of 2DCT into a 3D reconstruction.4.

3D printing has become increasingly utilized in the preoperative planning of clinical orthopaedics, trauma orthopaedics and other disciplines over the past decade [2]. 3D-printed models are readily accessible due to the wide availability of 3D printing techniques and 3D printers [2]. 3D printing contributes to a better understanding of the surgical approach, reduction and fixation of fractures, especially in complex fractures such as acetabular fractures [3, 4–6]. Zeng et al. [7] describe the combination of a 3D-printed model and a computer-assisted virtual surgical program for preoperative planning. This combination resulted in improved patient-specific preoperative planning. Furthermore, more accurate reduction and shorter operation times can be achieved [8, 9].

Mallepree et al. [10] concluded that the accuracy of a medical print was mostly influenced by scan parameters and not by the process of converting CT data into 3D prints. The process from scanning the patient to the final 3D-printed model will result in loss of data. However, it is unclear how a 3D-printed model relates to a human bone. To our knowledge, there is no literature that validates the accuracy of 3D-printed models in a preoperative planning strategy when applied to real human bones. The accuracy of 3D-printed models is important to pre-bend plates and fit of surgical guides. We have conducted a validation study in which we compare human cadavers with 3D-printed models to test the accuracy of 3D printing.

## Methods

### Study preparations

**Fig. 1:**
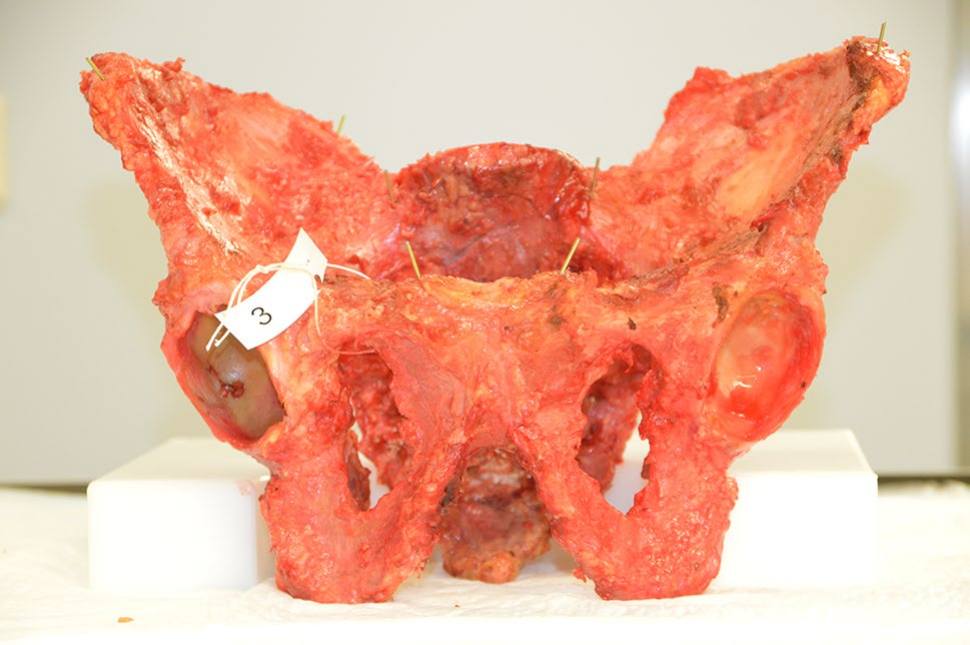
Shows dissected the pelvis cadaver number 3 with all five marker points

Three fresh frozen human cadavers were obtained from the Department of Anatomy. The pelvis, hands and feet were dissected and freed from all soft tissue exposing the bony structures. The ligamentous structures on the bone were left intact. Nine anatomic specimens − 3 pelvis, 3 hands and 3 feet—were used (Fig. 1).

**Fig. 2:**
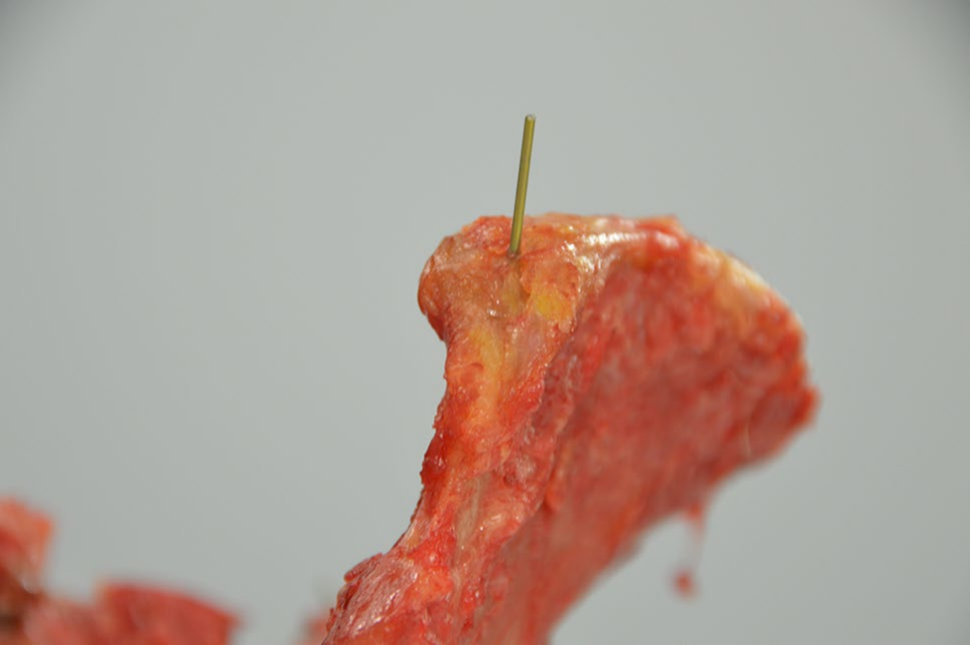
This is a close up of the dissected pelvis with one of the created marker point by titanium K-wires

Titanium Kirschner (K-) wires were inserted to mark anatomical landmarks (Fig. 2).

Pelvic landmarks were defined as the left and right: (1) tubercle on the pubic bone, (2) anterior superior iliac spine (ASIS), (3) posterior superior iliac spine (PSIS), (4) sacroiliac (SI) joint and (5) distance between the SI-joint and pubic bone on the right side of the pelvic bone.

**Fig. 3:**
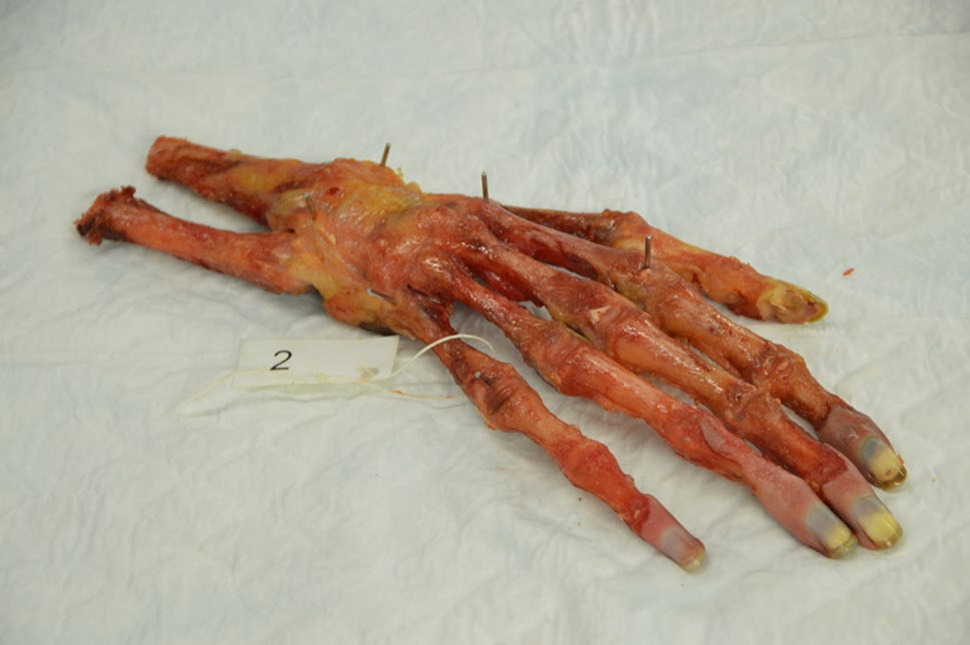
A cadaver of the hand with the marker points

Hand landmarks were defined as: (1) the radial styloid process and distal radioulnar articulation, (2) base and head of the second metacarpal bone and (3) base and head of the fifth metacarpal bone. Figure 3 shows a hand of a cadaver with these marker points.

**Fig. 4:**
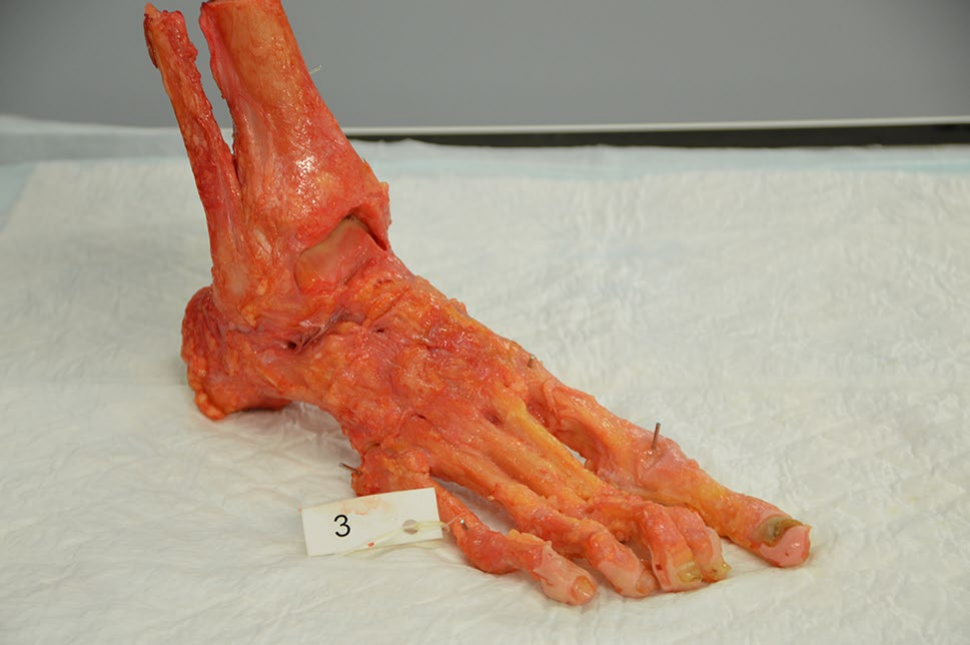
A cadaver of the foot with the marker points

Foot landmarks were defined as: (1) the distal medial malleolus, (2) between base and head of the first metatarsal bone and (3) base and head of the fifth metatarsal bone. Figure 4 shows a foot with the marker points. The distances between all landmarks were subsequently measured by two independent observers using a Vernier caliper. The point of intersection was defined as the intersection between bone and K wire.

### Process of creating 3D prints from CT data

In order to create a 3D print, a standard tessellation language (STL) file is needed. This is a specific file format used by 3D software to generate 3D prints. Converting CT scans in digital imaging and communication (DICOM) file format to STL occurs in three stages [11]: image acquisition, [12] image post-processing [13] and 3D printing.

### Image acquisition

The nine specimens were scanned using a Siemens Somatom Definition AS 64-slice CT (Siemens Healthcare, Forchheim, Germany). Slice thickness of 0.6 mm and soft reconstruction filters were used for our protocol in order to generate high resolution images and minimalize soft tissue image noise.

DICOM data of all cadavers were saved in Picture Archiving and Communication System (PACS). The two independent reviewers used the hospital’s integrated *Philips Intellispace Portal®* software to measure the distance between the markers in two-dimensional views.

### Image post-processing

The image post processing was divided into three phases:

#### Phase 1: creating a volume-rendered model of the object

**Fig. 5:**
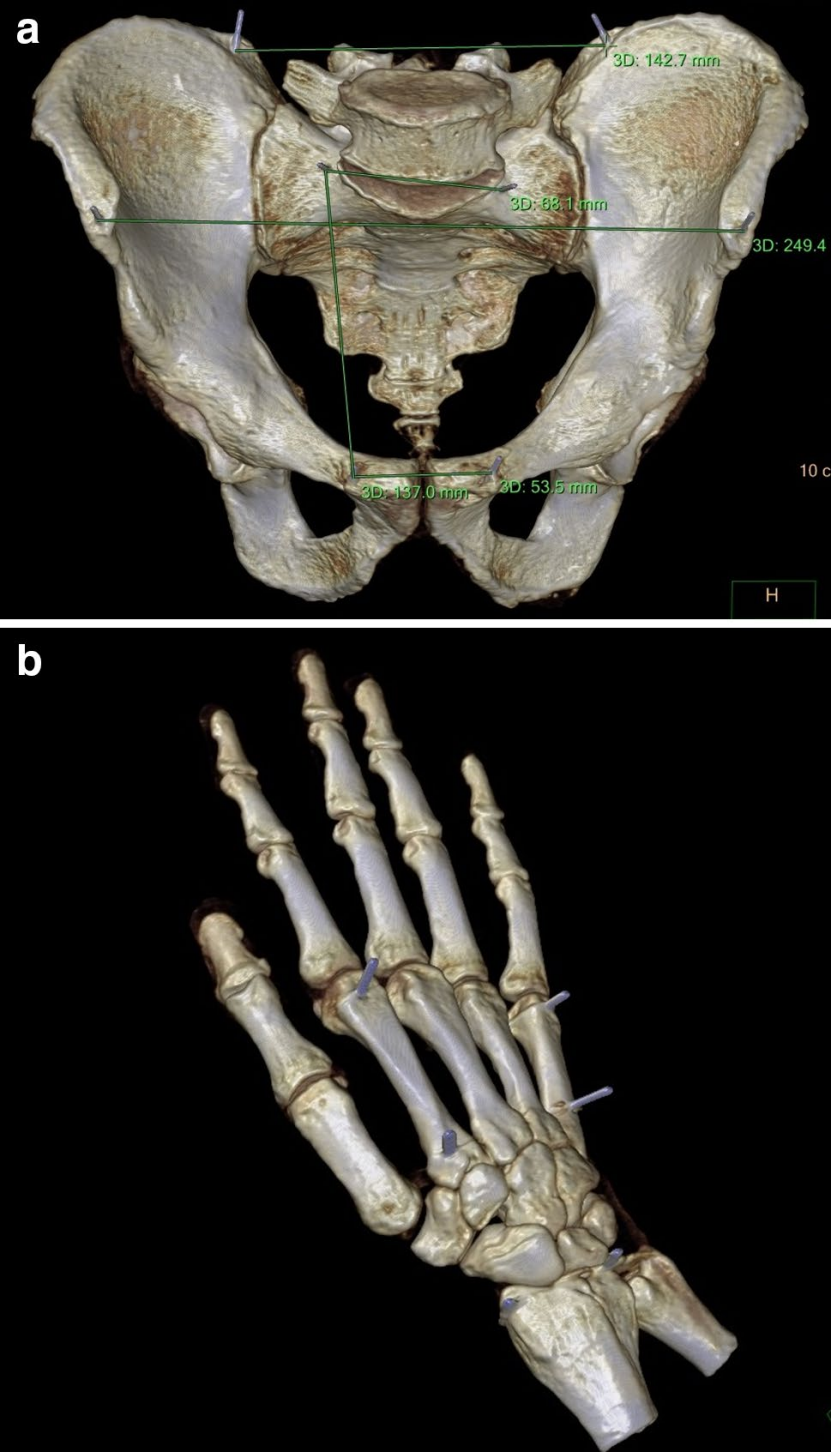
In this figure a 3D model of a pelvis after CT-scanning is seen with all measurements between the five marker points performed on the Philips Intellispace Portal

We used *Philips Intellispace Portal* software to volume render the DICOM data into 3D reconstructions and to ascertain measurements of the 3DCT landmarks by the two independent observers. Figure 5 shows a 3DCT of the pelvis and a hand with respectively the 5 and 3 anatomical landmarks.

**Fig. 6:**
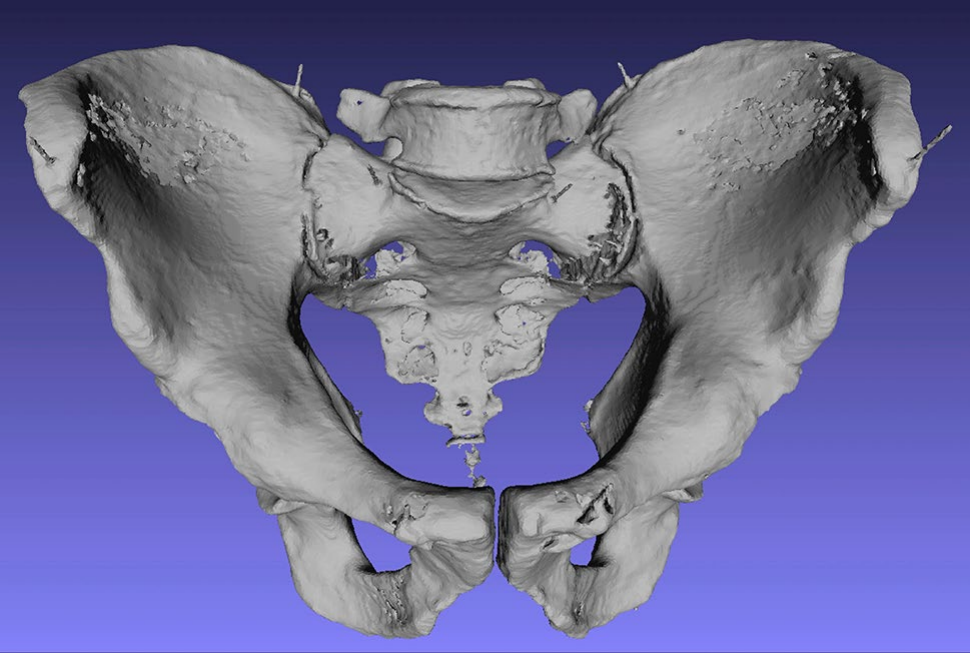
A view of the 3D model of a pelvis in the open software source Meshlab

#### Phase 2: cleaning of the model and creating an STL file from the volume-rendered model

The 3D reconstruction was digitally cleaned from all surrounding artifacts and remnants of the soft tissue in the Philips Intellispace Portal and then saved as an STL file. The landmarks in the STL file were measured by the two independent reviewers using Meshlab, an open-source program (Fig. 6).

#### Phase 3: importing the STL file in 3D print software and generating the print code

Our hospital uses both the *Makerbot Replicator Z18* (Makerbot Industries, USA)—a high end consumer extrusion 3D printer with a large build volume and the *Ultimaker 3* (Ultimaker B.V., the Netherlands) a desktop 3D printer with a dual extruder. These printers use Polylactic Acid (PLA), a thermoplastic polyester, to extrude the plastic on a build platform where it solidifies.

The print code (G-code) for the Makerbot was generated using *Simplify 3D* and the print code for the Ultimaker was generated using *Cura*. The following process settings were standardized: extruder temperature 215 °C, chamber temperature 24 °C, primary layer height 0.2 mm, infill 2% (the outer side of bone exists of cortical bone, therefore the model supports itself and less infill can be used), support infill 20%, maximum overhang without support 60%.

### 3D printing

**Fig. 7:**
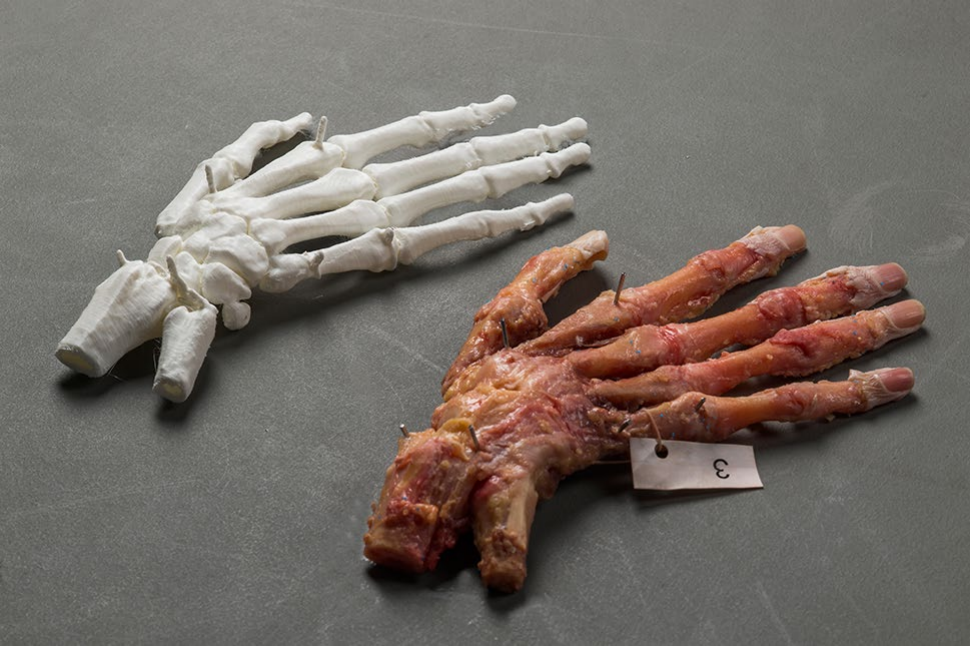
The cadaver hand with titanium K-wires maker points next to a 3D-printed model of the hand. The printed K-wires are clearly seen on the 3D-printed model

The 3D models of the cadavers were printed in a ratio of 1:1. A 3D-printed model of a hand and the cadaver hand can be seen in Fig. 7. The amount of material used, PLA and support, printing time and filament costs were also noted. The two independent observers measured all distances on all 3D-printed models.

### Two observers

All of the measurements described above were undertaken by two independent observers. In summary, they measured the distances between the anatomical landmarks on the human cadavers (cadaver), 2DCT (Port_2D), *3D reconstructions* (Port_3D), Meshlab (Mesh_3D) and 3D-printed models on the Ultimaker and Makerbot (Print_UM, Print_MB).

After 1 month, both observers were asked to measure all distances again to measure the inter-observer and intra-observer agreement. The distances between the K-wires on the fresh human cadavers were only measured once, because the cadavers had to be disposed of after two days.

### Data analysis

Descriptive statistics were calculated to provide an overview of the print process settings. Observer data were analyzed and expressed in terms of intra- and inter-observer agreement. We used Pearson’s correlation to calculate the correlation coefficient *r* and to analyse the relationship between the measurements of both observers.

The measurements between both 3D printers and cadavers were also expressed as a percentage of cadavers. A Paired Samples *T* test was used to compare the measurements between cadavers, 2DCT, 3DCT, Meshlab and both 3D printers. A *p* value of 0.05 was determined as significant. IBM SPSS Statistics 24 was used for the database (Statistical Package for the Social Sciences, Chicago, IL, USA).

## Results

Table 1 shows an overview of the print process settings of both 3D printers. The mean raw material costs for printing a pelvis, foot and hand were respectively 25, 6 and 4 euro for the Makerbot and 34, 13 and 6 euro for the Ultimaker.

**Table 1:** Printing characteristics

	Makerbot				Ultimaker			
	Building time in hours	Weight in grams	Support, %	Mean filaments costs in euro	Building time in hours	Weight in grams	Support, %	Mean filament costs in euro
Pelvis 1	92.00	613	32	25	97.00	710	45	34
Pelvis 2	56.00	392	53		76.00	588	59	
Pelvis 3	72.00	631	35		106.00	720	55	
Foot 1	23.50	140	24	6	30.26	270	50	13
Foot 2	24.00	116	38		22.30	227	51	
Foot 3	25.00	177	46		26.50	276	56	
Hand 1	11.20	70	29	4	13.50	111	58	6
Hand 2	16.00	70	23		14.50	118	49	
Hand 3	10.30	65	31		14.40	131	49	

Table 2 shows the correlation of the measurements between the observers—the inter-observer agreement. The Pearson correlation coefficient of 1 for each measurement shows that both observers had exact agreement in measuring the distances in all objects.

**Table 2:** Inter-observer agreements

	Pearson correlation inter-observer	*p* value
Cadaver	1.000	0.000
Port_2D	1.000	0.000
Port_3D	1.000	0.000
Mesh_3D	1.000	0.000
Print_UM	1.000	0.000
Print_MB	0.999	0.000
Port_2D_1	1.000	0.000
Port_3D_1	1.000	0.000
Mesh_3D_1	1.000	0.000
Print_UM_1	1.000	0.000
Print_MB_1	1.000	0.000

The intra-observer agreements of both observers are shown in Table 3. All Pearson correlations here too are 1, indicating absolute agreements for each observer.

**Table 3:** Intra-observer agreements

	Pearson correlation intra-observer_1	*p* value	Pearson correlation intra-observer_2	*p* value
Cadaver	–			–
Port_2D	1.000	0.000	1.000	0.000
Port_3D	1.000	0.000	1.000	0.000
Mesh_3D	1.000	0.000	1.000	0.000
Print_UM	1.000	0.000	1.000	0.000
Print_MB	1.000	0.000	1.000	0.000

Table 4 shows the mean distances of the objects measured on all modalities. The average distance in millimetres was calculated of each of the five marker points on the pelvis and three marker points on each foot and hand. For example; the mean distance measured on the cadaver of pelvis 1 was 129.90 mm, 2DCT: 130.17 mm, 3DCT: 130.40 mm, Meshlab: 130.07 mm, Ultimaker: 129.50 mm and Makerbot: 128.80 mm. Furthermore, in Table 4 the measurements of both 3D printers and the cadavers are compared and the difference in percentage between both modalities in calculated. In general, a decrease in measured distances can be seen in all specimens. The least decrease can be seen in “the 3D-printed pelvis 1”, − 0.3 and − 0.8% on respectively the Ultimaker and Makerbot when compared with cadaver Pelvis 1. The 3D model of “Hand 2” shows the most decrease, − 2.5 and − 3.2% on the Ultimaker and Makerbot when compared with cadaver hand 2.

**Table 4:** Mean measured distances in millimetres

	Cadaver	2D	3D	Mesh	UM (%)^a^	MB (%)^a^
Pelvis 1	129.90	130.17	130.40	130.07	129.55 (99.7)	128.80 (99.2)
Pelvis 2	136.60	137.07	136.78	137.03	135.25 (99.0)	135.10 (98.9)
Pelvis 3	129.40	129.58	129.42	129.39	128.00 (98.9)	127.25 (98.3)
Foot 1	61.17	62.35	60.97	62.06	61.00 (99.7)	60.42 (98.8)
Foot 2	68.83	69.00	68.56	68.98	67.42 (98.0)	67.67 (98.3)
Foot 3	54.50	54.27	53.97	54.53	53.25 (97.7)	52.92 (97.1)
Hand 1	45.17	45.22	45.32	44.93	44.67 (98.9)	44.25 (98.0)
Hand 2	44.00	43.91	43.67	44.48	42.92 (97.5)	42.58 (96.8)
Hand 3	38.83	39.33	39.62	38.74	38.42 (98.9)	38.33 (98.7)

*2D* 2-dimensional CT,* 3D* 3-dimensional CT,* Mesh* Meshlab,* UM* Ultimaker 3,* MB* Makerbot Replicator Z18

^a^The percentages given in the UM and MB column are the mean distances with reference to the measurements of the cadavers

Table 5 shows the *P* values of the differences in measurements between cadavers, 2DCT, 3DCT, Meshlab and both 3D printers. Most significant differences in measurements were found in the conversion from 3D file into a 3D print and between the cadaver and 3D-printed model from the Makerbot.

**Table 5:** A paired samples *t* test was used to compare the measurements between cadavers, 2DCT, 3DCT, Meshlab and both 3D printers

	Cadaver-2DCT	2DCT-3DCT	3DCT-Meshlab	Meshlab-UM	Meshlab-MB	Cadaver-UM	Cadaver-MB
Pelvis 1	0.658	0.317	0.591	*0.020*	*0.007*	0.720	0.330
Pelvis 2	0.382	0.222	0.646	*0.005*	*0.015*	0.055	*0.031*
Pelvis 3	0.551	0.597	0.961	*0.009*	*0.006*	0.130	*0.015*
Foot 1	0.092	0.081	0.207	0.190	0.065	0.423	*0.035*
Foot 2	0.486	0.298	0.263	*0.025*	*0.003*	*0.003*	*0.034*
Foot 3	0.395	0.391	0.161	0.091	*0.016*	0.130	*0.003*
Hand 1	0.701	0.096	0.299	0.568	0.558	0.423	0.368
Hand 2	0.774	0.332	0.121	0.049	*0.014*	0.096	0.161
Hand 3	0.293	0.212	0.073	0.539	0.632	0.497	0.597

A *p* value of 0.05 was determined as significant

## Discussion

3D-printed anatomical models have to be accurate, especially for pre-bending plates in complex fracture surgery [6, 14]. This validation study investigated the accuracy of our 3D printing process. To the best of our knowledge, the accuracy of 3D-printed anatomical models has not been investigated [5, 6, 15, 16]. In this study we validated the 3D printing process for our clinical setting.

The literature we reviewed on the clinical use of 3D printing in daily practice did not clarify the validation of their 3D printing process [17–20]. Mallepree et al. [10] reported in their study that accuracy of medical 3D models was mainly affected by scan parameters and not the printing process itself. However, our study highlighted different results. Table 4 highlights that step 1 (cadaver—2DCT) showed smaller differences in measured distances when compared with differences measured in step 4 (i.e. Meshlab—3D printers). In step 1, only “foot 1” showed a difference of > 1 mm between the measurements on the cadaver and 2DCT. In step 4, a difference of > 1 mm between the measurements using Meshlab and both printers was found in: pelvis 1, pelvis 2, pelvis 3, foot 1, foot 2 and hand 2 (Table 4). This observation is confirmed by the results in Table 5, which show a significant decrease in measured millimetres when both 3D printers are compared with Meshlab. No significant differences were found between the cadavers and CT and CT and Meshlab. Additionally, this table also shows that there are more significant differences between the cadaver and Makerbot, than between the cadaver and Ultimaker. Therefore, it seems that the Ultimaker is more accurate than the Makerbot.

**Fig. 8:**
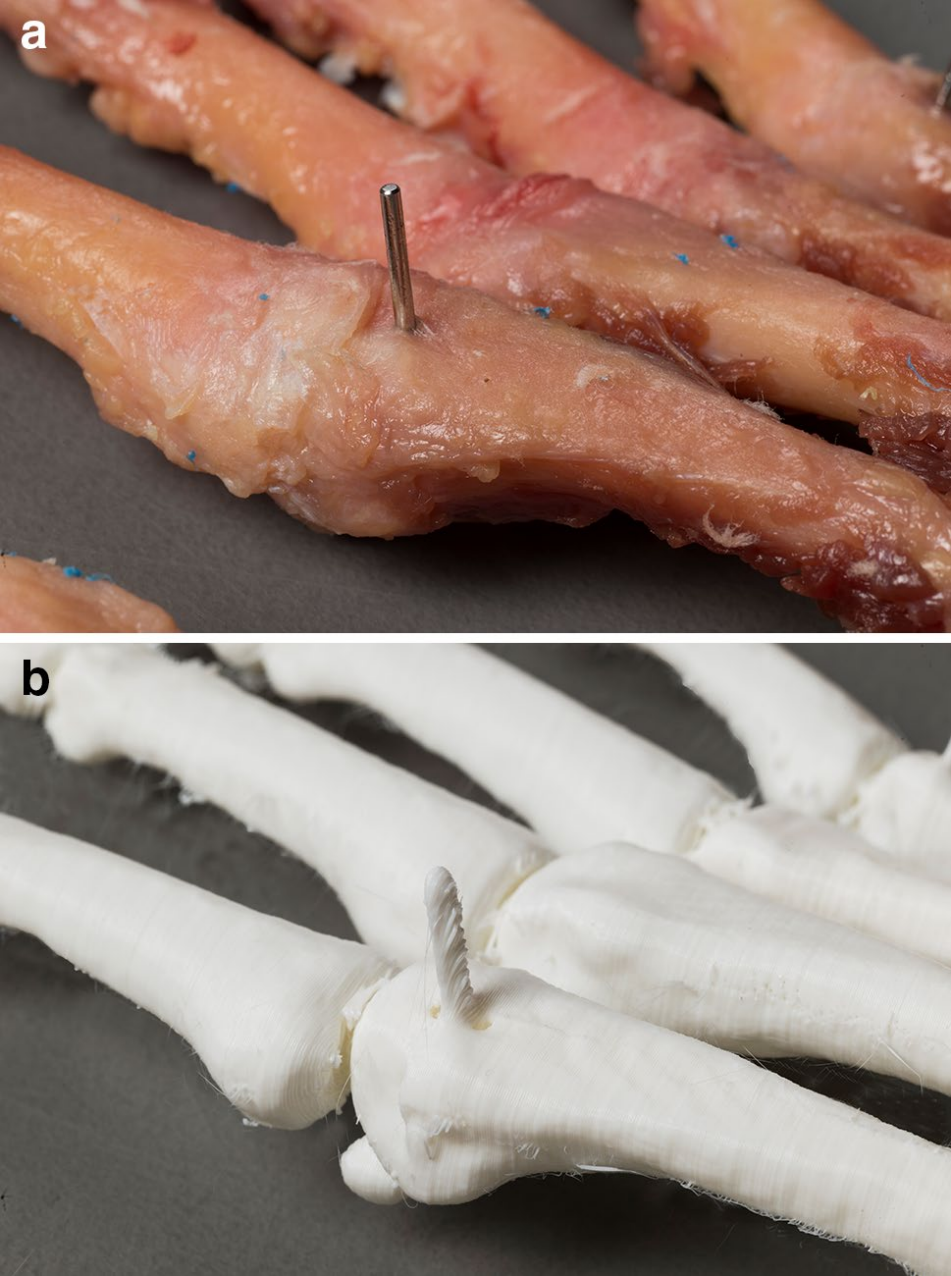
Difference between the titanium K-wires and 3D-printed pins

We measured a decrease in distance between the landmarks when comparing the actual 3D prints with the digital files. However, on closer inspection of the 3D-printed models, we noticed that the 3D-printed K-wires were more flattened than the actual K-wires (Fig. 8). This resulted in a shorter distance when measuring on 3D-printed models. A reason for this difference in shape could be that scanning titanium wires cause artifacts on the digital files which lead to small measurement errors on the 3D-printed objects.

Even though the differences between the cadavers and 3D-printed models are statistically significant (Table 5), we find the clinical importance less significant. We believe that these small differences will neither affect the position of the pre-bended plate nor the anatomy of the bone irrespective of the location or type of fracture.

Our study has some limitations. A small sample size (*n* = 9) was used. Nevertheless, a clear trend can be seen between the different modalities. In analysing 3D models, a volumetric analysis would be more accurate and is in fact the gold standard. We did not perform a volumetric analysis of the cadavers and the printed models, for this we would need a fluid displacement method [21, 22]. This in theory is an easy method but not feasible in our study. Utilising a fluid displacement model makes removal of all the soft tissue of the cadaver models essential. This however was not possible in our study design. Also, the PLA filament in use is permeable and hydrophilic. Therefore, a fluid displacement measurement comparison with a 3D-printed model would be unreliable as the model would absorb water. Another limitation of this study is that we used specific software to convert and modify the files and the results cannot be extrapolated to other software. Our results are only applicable for Philips software (Royal Philips N.V., the Netherlands) and the open source software we used. We only validated our 3D printing process and cannot say anything about 3D printing with other types of software and 3D printers.

## Conclusion

We can conclude that our 3D printing process results in accurate models suitable for preoperative workup. The Ultimaker 3 is slightly more accurate than the Makerbot Replicator Z18. Medical professionals must be aware that titanium can give artifacts on 3D-printed models. We advise that medical professionals should perform a study that tests the accuracy of their 3D printing process before using the 3D-printed models in medical practice.

## References

[1] Tack P, Victor J, Gemmel P, Annemans L (2016). 3D-printing techniques in a medical setting: a systematic literature review. BioMed Eng Online.

[2] Bagaria Vaibhav, Deshpande Shirish, Rasalkar Darshana D., Kuthe Abhay, Paunipagar Bhawan K. (2011). Use of rapid prototyping and three-dimensional reconstruction modeling in the management of complex fractures. European Journal of Radiology.

[3] Hurson C, Tansey A, O’donnchadha B, Nicholson P, Rice J, McElwain J (2007). Rapid prototyping in the assessment, classification and preoperative planning of acetabular fractures. Injury.

[4] Mitsouras D, Liacouras P, Imanzadeh A (2015). Medical 3D printing for the radiologist. Radiographics.

[5] Borrelli J, Goldfarb C, Catalano L, Evanoff BA (2002). Assessment of articular fragment displacement in acetabular fractures: a comparison of computerized tomography and plain radiographs. J Orthop Trauma.

[6] Kim HN, Liu XN, Noh KC (2015). Use of a real-size 3D-printed model as a preoperative and intraoperative tool for minimally invasive plating of comminuted midshaft clavicle fractures. J Orthop Surg Res.

[7] Huang H, Hsieh M, Zhang G (2015). Improved accuracy of 3D-printed navigational template during complicated tibial plateau fracture surgery. Australas Phys Eng Sci Med.

[8] Zeng C, Xing W, Wu Z, Huang H, Huang W (2016). A combination of three-dimensional printing and computer-assisted virtual surgical procedure for preoperative planning of acetabular fracture reduction. Injury.

[9] Chen XJ, Yuan JB, Wang CT, Huang YL, Kang L (2010). Modular preoperative planning software for computer-aided oral implantology and the application of a novel stereolithographic template: a pilot study. Clin Implant Dent Relat Res.

[10] Upex P, Jouffroy P, Riouallon G (2017). Application of 3D printing for treating fractures of both columns of the acetabulum: benefit of pre-contouring plates on the mirrored healthy pelvis. Orthop Traumatol Surg Res.

[11] Mallepree T, Bergers D (2009). Accuracy of medical RP models. Rapid Prototyp J.

[12] Mandrekar JN (2011). Measures of interrater agreement. Rapid Prototyp J.

[13] Landis JR, Koch GG (1977). The measurement of observer agreement for categorical data. Biometrics..

[14] Dai K, Yan M, Zhu Z, Sun Y (2007). Computer-aided custom-made hemipelvic prosthesis used in extensive pelvic lesions. J Arthroplasty.

[15] Wu XB, Wang JQ, Zhao CP (2015). Printed three-dimensional anatomic templates for virtual preoperative planning before reconstruction of old pelvic injuries: Initial results. Chin Med J (Engl).

[16] Chen Xu, Chen Xuanhuang, Zhang Guodong, Lin Haibin, Yu Zhengxi, Wu Changfu, Li Xing, Lin Yijun, Huang Wenhua (2017). Accurate fixation of plates and screws for the treatment of acetabular fractures using 3D-printed guiding templates: An experimental study. Injury.

[17] Izatt MT, Thorpe PLPJ, Thompson RG, Dâ€™Urso PS, Adam CJ, Earwaker JWS (2007). The use of physical biomodelling in complex spinal surgery. Eur Spine J.

[18] Modabber A, Legros C, Rana M, Gerressen M, Riediger D, Ghassemi A (2012). Evaluation of computer-assisted jaw reconstruction with free vascularized fibular flap compared to conventional surgery: a clinical pilot study. Int J Med Robot.

[19] Ayoub N, Ghassemi A, Rana M, Gerressen M, Riediger D, Holzle F (2014). Evaluation of computer-assisted mandibular reconstruction with vascularized iliac crest bone graft compared to conventional surgery: a randomized prospective clinical trial. Trials.

[20] Wilde F, Winter K, Kletsch K, Lorenz K, Schramm A (2014). Mandible reconstruction using patient-specific pre-bent reconstruction plates: comparison of standard and transfer key methods. Int J Comput Assist Radiol Surg.

[21] Partik BL, Stadler AF, Schamp SF (2002). 3D versus 2D ultrasound: Accuracy of volume measurement in human cadaver kidneys. Invest Radiol.

[22] Bakker Jeannette, Olree Marco, Kaatee Robert, de Lange Eduard E, Beek Ferdinand J.A (1998). In vitro measurement of kidney size: comparison of ultrasonography and MRI. Ultrasound in Medicine & Biology.

